# Cholesterol Overload: Contact Sites to the Rescue!

**DOI:** 10.1177/2515256419893507

**Published:** 2019-12-05

**Authors:** Carlos Enrich, Carles Rentero, Thomas Grewal, Clare E. Futter, Emily R. Eden

**Affiliations:** 1 Departament de Biomedicina, Unitat de Biologia Cel·lular, Facultat de Medicina i Ciències de la Salut, Universitat de Barcelona, Spain; 2 Centre de Recerca Biomèdica CELLEX, Institut d’Investigacions Biomèdiques August Pi i Sunyer, Barcelona, Spain; 3 School of Pharmacy, Faculty of Medicine and Health, University of Sydney, New South Wales, Australia; 4 UCL Institute of Ophthalmology, London, UK

**Keywords:** ER-lysosome contact sites, cholesterol transport, NPC1, Annexin A6, TBC1D15

## Abstract

Delivery of low-density lipoprotein-derived cholesterol to the endoplasmic reticulum (ER) is essential for cholesterol homeostasis, yet the mechanism of this transport has largely remained elusive. Two recent reports shed some light on this process, uncovering a role for Niemann Pick type-C1 protein (NPC1) in the formation of membrane contact sites (MCS) between late endosomes (LE)/lysosomes (Lys) and the ER. Both studies identified a loss of MCS in cells lacking functional NPC1, where cholesterol accumulates in late endocytic organelles. Remarkably, and taking different approaches, both studies have made a striking observation that expansion of LE/Lys-ER MCS can rescue the cholesterol accumulation phenotype in NPC1 mutant or deficient cells. In both cases, the cholesterol was shown to be transported to the ER, demonstrating the importance of ER-LE/Lys contact sites in the direct transport of low-density lipoprotein-derived cholesterol to the ER.

Cholesterol is essential for membrane organization and the synthesis of steroid hormones, bile acids, and vitamin D. Hence, cells have developed sophisticated machinery to coordinate cholesterol homeostasis. Internalized dietary low-density lipoprotein (LDL)-derived cholesterol in late endosomes (LE) is delivered to the endoplasmic reticulum (ER), which senses cholesterol levels, enabling downregulation of cholesterol synthesis. Niemann Pick type-C1 protein (NPC1) is considered the major cholesterol transporter in LE/lysosomes (Lys), and loss-of-function NPC1 mutations cause NPC disease, which is associated with cholesterol accumulation in LE. Using yeast NPC proteins as a model, a recent study from Winkler et al. (2019) provides novel insight into how cholesterol is integrated into the lysosomal limiting membrane. However, the mechanisms underlying onward transport of cholesterol from the cytoplasmic leaflet of LE/Lys for redistribution to other organelles are still not well understood. In this context, membrane contact sites (MCS) have recently gained attention as platforms to facilitate non-vesicular cholesterol transport between LE/Lys and other organelles. Two recent independent studies now provide experimental evidence that induced MCS formation between LE/Lys and the ER can bypass NPC1 loss-of-function in directing LDL-cholesterol transfer to the ER.

Although clearly visible on early electron micrographs, only during the past decade have MCS between ER and LE/Lys gained recognition as important hubs for extensive interorganellar cross-talk. However, the physiological relevance and molecular machinery connecting these two compartments at MCS has been hard to decipher. It is now known that ER-endosome contact sites orchestrate several coordinated events important in shaping the endocytic pathway, including endosome biogenesis and cargo sorting, endosome positioning, motility and fission, calcium signaling and traffic of lipids, including cholesterol (Cabukusta and Neefjes, 2018; Ridgway and Zhao, 2018). Cholesterol is actively transported in both directions at the ER-LE/Lys interface, with the directionality and rate of transport being central for cholesterol homeostasis, membrane biogenesis, and proper functioning of mammalian cells.

On close inspection, the ER consists of an extensive 3-dimensional network of tubule-cisternae membranes that we now know are “in touch” with most other cellular compartments. This has greatly changed the paradigm of interorganellar communication and several proteins are engaged in lipid transfer at the interface of opposing organelles. Intriguingly, some of these factors not only enable dynamic transport of lipids between compartments but are also implicated as tethers, maintaining close contact between organelles. Findings from our laboratories have now identified a subset of proteins that participate in MCS formation and function to facilitate cholesterol transfer across MCS, restoring cholesterol homeostasis in NPC1 mutant cells (Figure 1).

Firstly, the study by Höglinger et al. (2019) uncovered a novel role for NPC1 as a MCS tethering protein. At MCS, NPC1 interacts with ER-localized Gramd1b and mediates MCS formation. Gramd1 proteins transfer sterols via a Steroidogenic Acute Regulatory protein-related lipid Transfer (StART)-like domain and Gramd1b efficiently transports cholesterol. Gramd1b senses cholesterol-rich membranes and was previously found at MCS between the ER and plasma membrane (Sandhu et al., 2018). Höglinger et al. also demonstrated Gramd1b interaction with NPC1 at ER-lysosome contact sites, dependent on the presence of LDL-derived cholesterol in the endocytic pathway. These findings support a model of Gramd1b redistribution to establish contacts with lysosomes when increased amounts of LDL-cholesterol are endocytosed, promoting interaction with NPC1 to facilitate inter-organellar transport of cholesterol to the ER. The importance of this interaction to stabilize the MCS was highlighted by Gramd1b depletion reducing ER-LE/Lys MCS to a similar extent as in NPC1-deficient cells. Moreover, localization of NPC1 on LE/Lys-ER contacts was strongly reduced upon Gramd1b depletion. This study indicates roles for NPC1 beyond cholesterol transport, acting as a tether to stabilize MCS that provide platforms for cholesterol transfer through interaction with Gramd1b at LE/Lys-ER contact sites, and possibly in a similar manner at other cellular sites. Overexpression of a late endosomal oxysterol-binding protein homologue (ORP1L) mutant that cannot sense or transport sterol, but acts as an artificial tether, can expand ER-lysosome contacts through constitutive interaction with VAP on the ER. Remarkably, MCS expansion by sterol-binding mutant ORP1L reversed LE/Lys-cholesterol accumulation in NPC1 mutant cells, indicating that alternative transport routes across MCS can compensate for NPC1 deficiency.

At the cellular level, NPC1 loss-of-function and consequent accumulation of cholesterol and other lipids is accompanied not only by dysfunctional LE/Lys but also impaired oxidative phosphorylation and disrupted assembly of respiratory supercomplexes in mitochondria, thought to be caused by elevated mitochondrial cholesterol (Solsona-Vilarrasa et al., 2019). Höglinger et al. found that under conditions of elevated lysosomal cholesterol, when ER contact was reduced by depletion of either Gramd1b or NPC1, lysosomes formed tight connections with mitochondria. This expansion of lysosome-mitochondria MCS may explain the increased mitochondrial cholesterol observed in NPC1-deficient cells. Moreover, just as the rise in mitochondrial cholesterol is StARD3-dependent in NPC (Charman et al., 2010), StARD3 was also essential for the expanded lysosome-mitochondria MCS population.

In the study by Meneses-Salas et al. (2019), depletion of the scaffolding protein AnxA6 alleviated LE-cholesterol accumulation in NPC1 mutant cells. Mechanistically, AnxA6 associates with cholesterol-rich endosomes in NPC1 mutants and interacts with a TBC family member, TBC1D15, a Rab7-GTPase activating protein (GAP). This interaction of AnxA6 and TBC1D15 in the LE-compartment promotes TBC1D15-mediated inactivation of Rab7. Consequently, depletion of either AnxA6 or TBC1D15 increased Rab7-GTP levels and increased mobility and peripheral distribution of LE. Most noticeably, and similar to the rescue of the NPC1 phenotype upon ectopic Rab7 overexpression (Choudhury et al., 2002), upregulated endogenous Rab7-GTP levels in NPC1 mutant cells were sufficient to promote LE/Lys-cholesterol egress, followed by an enhanced neutral lipid accumulation in lipid droplets in an Acyl-CoA: Cholesterol Acyltransferase (ACAT)-dependent manner, reflecting increased LE/Lys to ER cholesterol transport. Importantly, electron microscopy revealed extensive membrane contacts between LE/Lys and the ER in wild-type cells, in contrast to NPC1 mutants which displayed low MCS numbers between these two organelles. However, on silencing AnxA6, a significant increase of MCS between LE and the ER was observed in NPC1 mutant cells. Given the involvement of Rab7 in the formation of LE/Lys contacts with other organelles (Raiborg et al., 2015; Wong et al., 2018) and AnxA6 in recruiting TBC1D15 to regulate Rab7 hydrolysis, this strongly suggests cholesterol transfer from LE to the ER via Rab7-dependent MCS formation in AnxA6-depleted NPC1 mutant cells. This study identifies AnxA6 as a novel gatekeeper of the cellular machinery that controls distribution of LE-cholesterol via regulation of a Rab7-GAP and MCS formation.

Within this context, it is worth noting that another member of the annexin family, AnxA1, can serve as a tether, together with S100A11 proteins, in the formation of ER-endosome contacts in epidermal growth factor stimulated HeLa cells (Eden et al., 2016), indicating differential roles for different annexins in the coordination of MCS between LE/Lys and the ER. Moreover, while AnxA6-regulated contacts mediate cholesterol transfer from LE/Lys to the ER, AnxA1-tethered MCS were implicated in ORP1L/Vap-dependent cholesterol transport in the opposite direction, from the ER to endosomes (Eden et al., 2016). Interestingly ORP1L also regulates transport of LDL-derived cholesterol to the ER (Zhao and Ridgway, 2017), suggesting functionality in bidirectional cholesterol transport between the ER and LE/Lys. ER to LE/Lys sterol transport has been proposed to cause constitutive mTORC activation in NPC1-deficient cells because of the lack of opposing Lys to ER transport (Lim et al., 2019). In contrast to our findings, this study reported increased ER-LE contact in NPC1 patient fibroblasts, possibly reflecting differential expression of tethers/transporters (CHO cells do not express ORP1L), functional differences in NPC1 mutations, or an additional underlying genetic difference. Meneses-Salas et al. found that restoration of LDL-cholesterol transport to the ER in NPC1 mutant cells by AnxA6 depletion was dependent on StARD3, which like ORP1L, is a VAP-binding sterol transfer protein on LE/Lys that mediates ER to endosome cholesterol transport (Wilhelm et al., 2017). However, StARD3 has not previously been implicated in LE/Lys to ER cholesterol transport and although its overexpression expands LE/Lys-ER MCS through VAP interaction, loss of contact on StARD3 depletion has not been reported. It is possible that there is tethering redundancy at these MCS and that the requirement for endogenous StARD3 in LE/Lys to ER sterol transport only becomes apparent in the absence of functional NPC1 or ORP1L. These findings suggest that, like ORP1lL, StARD3 may also be able to mediate bidirectional sterol transfer between the ER and endosomes. In addition, StARD3 promotes contact between cholesterol-laden lysosomes and mitochondria in NPC1-deficient cells, likely as an alternative route for cholesterol egress from LE/Lys (Hoglinger et al., 2019).

Together, Höglinger and Meneses-Salas describe for the first time a strongly diminished number of MCS between LE/Lys and ER in NPC1 mutant cells. Most excitingly, the induction of MCS formation between these two organelles can bypass the NPC1 mutation, directing LDL-cholesterol transfer to the ER, thereby rescuing the NPC1 mutant phenotype. These studies highlight the functioning of MCS as novel therapeutic targets for the design of new therapies aiming to overcome cholesterol transport defects in NPC1 disease.

## Figures and Tables

**Figure 1:**
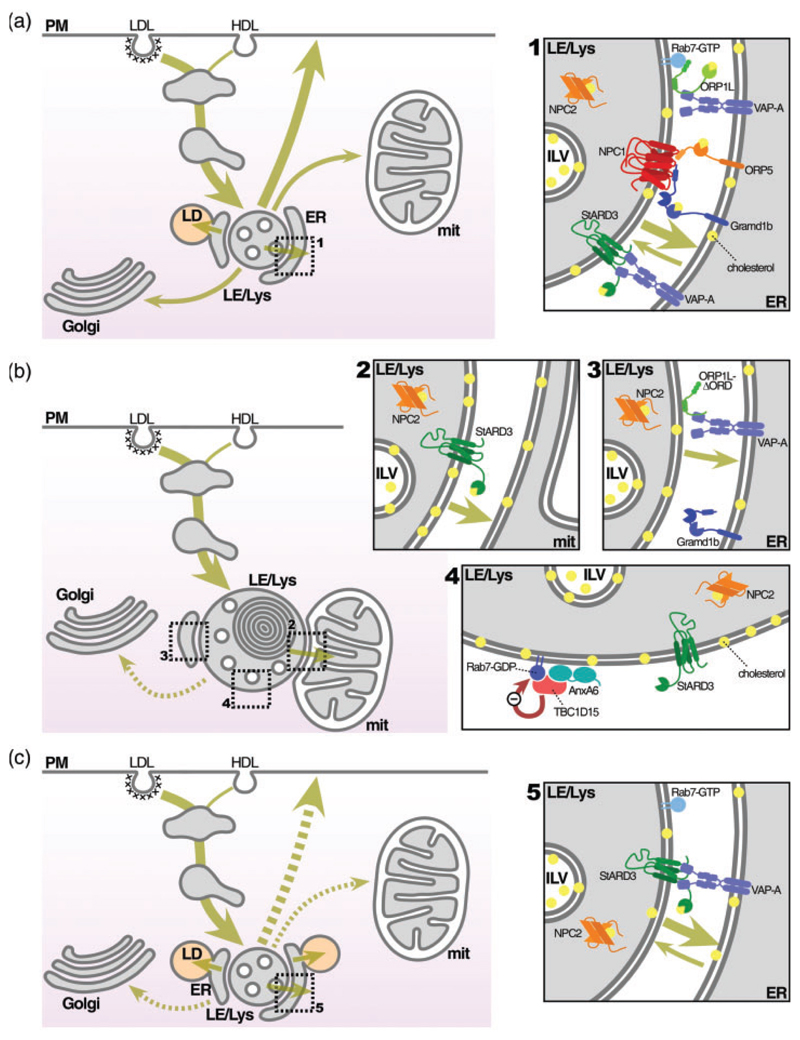
Proposed MCS interplay between LE/Lys with either ER or mitochondria for cholesterol egress from LE/Lys of NPC1 mutant cells. (a) Under normal conditions (wild type), LDL-cholesterol is internalized and delivered to LE/Lys. From there, LDL-derived cholesterol (yellow) is distributed/sorted mainly to the PM and the ER. Other less demanding destinations are mitochondria and the Golgi apparatus. In this setting, MCS between LE and ER are abundant and a significant number of LD for the storage of LDL-derived cholesteryl esters can be observed. The inset 1 illustrates some of the molecular partners on both sides of MCS, including Gramd1b which interacts with NPC1. (b) In the absence of NPC1, NPC1-tethered lysosome-ER contact sites are lost and cholesterol accumulates in the lysosome. Under these conditions, StARD3 mediates extended lysosome contact sites and cholesterol exchange with mitochondria (inset 2). Inset 4 shows the lack of NPC1 in the LE/Lys membrane and the untethering effect of AnxA6, which is recruited to LE/Lys in NPC1 mutant cells, promoting TBC1D15-mediated Rab7 inactivation (Rab7-GDP). As described by Höglinger et al. (2019), creating an artificial tether by overexpression of an ORP1L mutant, that cannot sense or transport sterol, but only expand ER-lysosome contacts, can establish cholesterol transport routes across MCS (inset 3). (c) Cholesterol transport to the ER in NPC1 mutant cells is restored upon AnxA6 depletion. Inset 5 illustrates the role of StARD3 transferring cholesterol from LE/Lys to the ER (in the direction of the cholesterol concentration gradient) across MCS. AnxA6 depletion leads to loss of TBC1D15-mediated Rab7 inactivation and consequently, upregulated Rab7 GTP levels. The implication of this increased Rab7-GTP in the formation or functioning of MCS is unknown. AnxA6 = Annexin A6; ER = endoplasmic reticulum; HDL = high-density lipoprotein; ILV = intraluminal vesicle; LD = lipid droplet; LDL = low-density lipoprotein; LE/Lys = late endosomal/lysosomal compartment; MCS = membrane contact sites; NPC1 = Niemann-Pick Type C1; PM = plasma membrane; mit = mitochondria.
